# Targeted Surface Expression of an Exogenous Antigen in Stably Transfected *Babesia bovis*

**DOI:** 10.1371/journal.pone.0097890

**Published:** 2014-05-19

**Authors:** Jacob M. Laughery, Donald P. Knowles, David A. Schneider, Reginaldo G. Bastos, Terry F. McElwain, Carlos E. Suarez

**Affiliations:** 1 Program in Vector-Borne Diseases, Department of Veterinary Microbiology and Pathology, Washington State University, Pullman, Washington, United States of America; 2 Animal Disease Research Unit, Agricultural Research Service, United States Department of Agriculture, Pullman, Washington, United States of America; 3 Paul G. Allen School for Global Animal Health, College of Veterinary Medicine, Washington State University, Pullman, Washington, United States of America; Obihiro University of Agriculture and Veterinary Medicine, Japan

## Abstract

*Babesia bovis* is a tick-borne intraerythocytic protozoan responsible for acute disease in cattle which can be controlled by vaccination with attenuated *B. bovis* strains. Emerging *B. bovis* transfection technologies may increase the usefulness of these live vaccines. One use of transfected *B. bovis* parasites may be as a vaccine delivery platform. Previous transfection methods for *B. bovis* were limited by single expression sites and intracellular expression of transfected antigens. This study describes a novel transfection system in which two exogenous genes are expressed: one for selection and the other for a selected antigen designed to be delivered to the surface of the parasites. The strategy for duplicating the number of transfected genes was based on the use of the putative bidirectional promoter of the *B. bovis* 1.4 Kb *ef-1α* intergenic region. The ability of this region to regulate two independent expression sites was demonstrated using a luciferase assay on transiently transfected *B. bovis* parasites and then incorporated into a stable transfection plasmid to control independent expression of the selectable marker GFP-BSD and another gene of interest. A chimeric gene was synthetized using sequences from the protective B-cell epitopes of *Rhipicephalus microplus* tick antigen Bm86 along with sequences from the surface exposed *B. bovis* major surface antigen-1. This chimeric gene was then cloned into the additional expression site of the transfection plasmid. Transfection of the *B. bovis* Mo7 strain with this plasmid resulted in stable insertion into the *ef-1α* locus and simultaneous expression of both exogenous genes. Expression of the Bm86 epitopes on the surface of transfected merozoites was demonstrated using immunofluorescence analyses. The ability to independently express multiple genes by the inclusion of a bidirectional promoter and the achievement of surface expression of foreign epitopes advances the potential of transfected *B. bovis* as a future vaccine delivery platform.

## Introduction

*Babesia bovis* is a tick borne intraerythocytic protozoan parasite that causes fatal acute and persistent disease in cattle and is a threat to cattle industries worldwide [1], [2], [3], [4]. Cattle that survive the acute phase of the disease become persistently infected and serve as a source of parasite transmission through tick vectors, such as *Rhipicephalus (Boophilus) microplus*. The strategies available to control bovine babesiosis include tick control by acaricides and vaccines, use of babesiacides, and vaccination of cattle with live *B. bovis* attenuated vaccine strains. Live attenuated vaccines are able to elicit strong, long term immune responses in cattle [2], [4], [5], [6]. The protection afforded by these vaccines is in part due to the ability of *Babesia* parasites to establish persistent infections with strong and continuous stimulation of the immune system of the vaccinated cattle. Taking advantage of these characteristics, we propose to expand the scope of current *B. bovis* live vaccines by developing an antigen delivery platform based on the use of transfected *B. bovis* parasites. Development of such vaccines is supported by the ability of transfected *B. bovis* to cause mild acute and persistent disease in cattle while remaining genetically stable [7].

Currently available *B. bovis* transfection systems use a single promoter controlling expression of an exogenous gene needed for selection of the transfected parasites [8], [9]. Ideally, a transfection-based antigen delivery platform would benefit from independent expression of the antigen of interest from the selectable marker. Achieving this feature would require the use of at least two independent promoters. The *B. bovis* elongation factor*-1α* (*ef-1α*) locus contains two identical head to head genes that are separated by a 1.4 Kb intergenic region (IG). It was previously shown that the two halves of the IG-regions, termed “A” and “B”, are independently able to function as promoters *in vitro* [9]. The complete 1.4 Kb *ef-1α* IG region was proposed to regulate bidirectional transcription of the two elongation factor open reading frames (ORFs) in *B. bovis* parasites [9], but its functional properties have not been fully characterized. Using a bidirectional promoter in the transfection plasmid would simplify the configuration and construction of the transfection plasmid, where the size of the intervening sequences may affect the efficiency of the homologous recombination events required for the insertion of the transfected genes.

Antigen candidates that may be delivered by a transfected *B. bovis* vaccine include protective proteins from its tick vectors. Theoretically, the inclusion of antigens able to induce protection against vector ticks could enhance control of *B. bovis* and other babesial species, as well as other hemoparasites transmitted by the same vector. Bm86, a concealed glycoprotein identified in the apical membrane of the tick midgut epithelium, has been shown to elicit a protective immune response against *R. (B.) microplus* in vaccinated cattle and reduce the intensity of tick infestation, egg laying capacity, and fertility [10], [11], [12]. Furthermore, B-cell epitope mapping of Bm86 has identified specific regions of the molecule that are able to elicit protective antibodies against *R. microplus* ticks when used as peptide-based vaccines in cattle [13], [14], [15]. Although recombinant Bm86 vaccines have been commercially produced for tick control [10], [11], [16], these vaccines are not fully effective and might not be practical for use in extensive cattle operations due to the need for frequent booster immunizations, a consequence of their relatively low immunogenicity and short term effectiveness [17], [18], [19]. However, a vaccine system that naturally and continuously boosts cattle with Bm86 has the potential to overcome these limitations and contribute to improved control of these harmful parasites. Thus, selected Bm86 protective B-cell epitopes may constitute appropriate candidates for expression of a foreign antigen in a transfected *B. bovis* delivery platform.

In addition, an effective antigen delivery platform should ideally be able to present the molecules of interest in a highly antigenic configuration. Some antigens exposed on the surface of *B. bovis* are known to be highly immunodominant. The *B. bovis* merozoite surface antigen 1 (MSA-1) is known to be an abundant, strongly antigenic, surface exposed, GPI anchored glycoprotein that is able to elicit early and strong humoral immune responses [20], [21]. MSA-1 also contains a leader sequence [22], [23], [24] that likely routes the antigen to the surface of the parasite where it is deployed and/or secreted. A neutralizing monoclonal antibody, termed Babb35 recognizes a B-cell epitope located in the highly antigenic hypervariable region of MSA-1 [20], [21], [24], [25]. It might be possible to enhance immunogenicity of foreign epitopes in transfected *B. bovis* parasites by taking advantage of the features of MSA-1 in a chimeric transfection construct.

In this study we describe a novel *B. bovis* transfection system based on a bidirectional promoter able to regulate independent and simultaneous expression of two distinct exogenous genes. In addition, we demonstrate surface merozoite expression of selected Bm86 B-cell epitopes by a chimeric gene containing *msa-1* and *Bm86* sequences in stably transfected *B. bovis*.

## Results

### 2.1 Bidirectional promoter activity of the full size *B. bovis* ef-1α intergenic region

**Figure 1:**
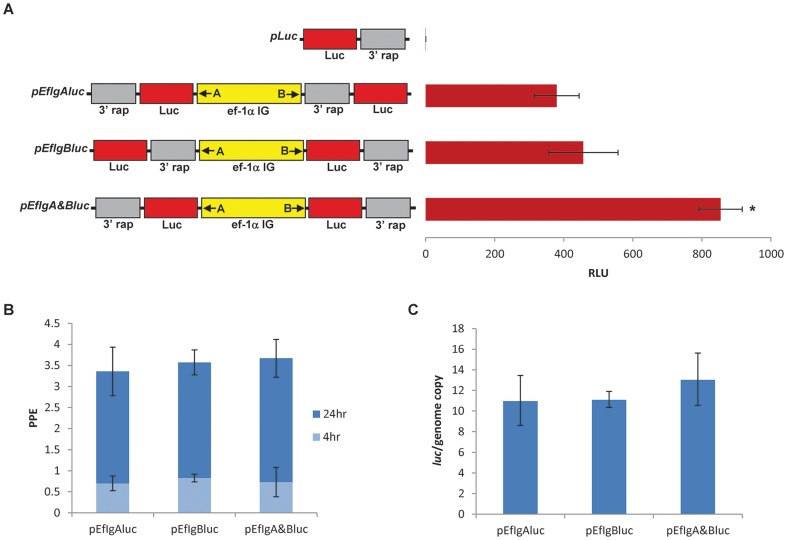
Full size *B. bovis ef-1α* IG region acts as bidirectional promoter. **A**. Plasmids *pLuc* (no promoter control), *pEfIgAluc* (one functional luciferase gene under transcriptional control of the Ig-A promoter), *pEfIgBluc* (one functional luciferase gene under transcriptional control of the Ig-B promoter), and *pEfIgA&Bluc* (two functional luciferase genes under transcriptional control of the Ig-A and Ig-B promoters) are schematically represented together with a chart showing luciferase activity generated by transiently transfected Mo7 *B. bovis*. The 1.4 kb *ef-1α* IG is shown in yellow with “A” and “B” orientation of the promoter as indicated with an arrow. The luciferase genes are represented in red and the *3′ rap* region used to stop transcription is indicated in grey. Data on the right represent luciferase expression in *B. bovis* lysates at 24 hours after transfection from each respective transfection plasmid. Luciferase activity is expressed as relative luciferase units (RLU). Error bars represent standard deviation of the mean for three independent experiments. Asterisk represents significant differences among luciferase activities on lysates from parasites transfected with plasmids containing a promoter. (*p*<0.05). **B**. Percentage of parasitized erythrocytes (PPE) of transfected parasite lines transfected with *pEfIgAluc, pEfIgBluc*, and *pEfIgA&Bluc*, obtained at 4 and 24 hours post-electroporation. Error bars represent standard deviation of the mean for three independent experiments. **C**. Quantification of the number of plasmid copies per MSA-1 gene copy (luc/genome copy), considering that the *B. bovis* genome contains a single MSA-1 gene, calculated 24 hours post-electroporation using quantitative PCR. Each cell line transfected with *pEfIgAluc, pEfIgBluc*, and *pEfIgA&Bluc* are shown. Error bars represent standard deviation of the mean for three independent experiments.

Three plasmid constructs were generated containing functional luciferase genes on the “A” end only (*pEfIgAluc*), “B” end only (*pEfIgBluc*), and on both the “A” and “B” ends (*pEfIgA&Bluc*) of the full size 1.4 kb *ef-1α* IG region (Fig. 1A). The three plasmids, together with negative control plasmid *pLuc* containing the luciferase gene but lacking a promoter, were transiently transfected into *B. bovis* Mo7 parasites and then analyzed for luciferase activity (Fig. 1A). No significant luciferase activity was detected in lysates from the *pLuc* transfected parasites. In contrast, significant luciferase activity levels were detected in lysates from parasites transfected with the plasmids where the functional luciferase gene was cloned immediately downstream of either the “A” (*pEfIgAluc*) or “B” (*pEfIgBluc*) *ef-1α* promoter ends in their correct orientation (Fig. 1A), demonstrating the presence of independent promoters located at both ends of the *ef-1α* IG region. Consistently, a significant increase in luciferase activity was found when functional luciferase genes were cloned at both ends (*pEfIgA&Bluc*) of the full size *ef-1α* IG (p<0.05) (Fig. 1A). Transfected *B. bovis* growth curves and real-time PCR quantification of the amount of plasmids per genome analyzed twenty four hours after transfection demonstrated similar amounts of transfected cells and ratios among plasmid per genomic copies in each of the transfections (Fig. 1B and C). Collectively, these data supports that the differences observed in luciferase expression between the three luciferase plasmids tested are not due to differential post-electroporation parasite viability, nor a result of variable efficiencies of transfection, and are consistent with the presence of a bidirectional promoter in the 1.4 Kb *ef-1α* IG region.

### 2.2 Stably transfected *B. bovis* parasites express the gfp-bsd and Bm86-msa-1 chimera genes

**Figure 2:**
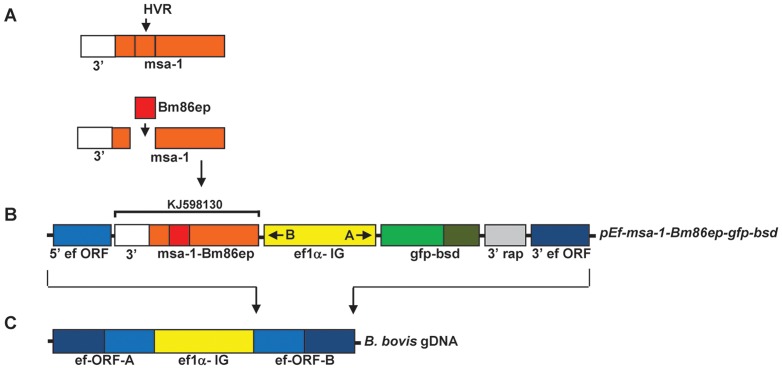
Construction of the stable transfection plasmid *pEf-msa-1-Bm86ep-gfp-bsd*. **A**. Schematic description and design of the *msa-1-Bm86ep* synthetic chimera gene using the sequences encoding for the *Bm86* epitopes indicated by the red bar replacing the hyper-variable coding region (HVR) of *msa-1*, indicated in orange. The white bar represents a 3′ region of *msa-1* used to control stop of transcription **B**. The schematic components of stable transfection plasmid *pEf-msa-1-Bm86ep-gfp-bsd* are shown. The dark and light blue bars represent the 5′ and 3′ regions of the *ef-1α* ORF, the yellow bars represent the 1.4 kbp *ef-1α* IG region, the composite orange and red bar represent the *msa-1-Bm86ep* chimera with the 3′ *msa-1* as shown under the GeneBank accession number for the compiled sequence (KJ598130), the light and dark green bars represent *gfp* and *bsd* respectively, and the grey bar represents a 3′ region of *rap-1* used to control stop of transcription. **C**. The *ef-1α* locus of the *B. bovis* genome is represented and the arrows indicate where the homologous recombination event of the transfection plasmid is designed to occur.

A plasmid was then designed for stable transfection of *B. bovis* parasites that takes advantage of the bidirectional promoter function identified in the 1.4 Kb *ef-1α* IG region. A chimeric gene encoding for a fusion of MSA-1 and selected Bm86 sequences (*msa-1-Bm86ep*) was synthetically produced, where the 299 bp hypervariable region of the *msa-1* gene was replaced with a 351 bp fragment encoding for Bm86 B-cell epitopes (Fig. 2A) (GenBank accession number: KJ598130). This chimera gene was cloned in the “B” promoter expression site of the transfection vector, while the *gfp-bsd* selectable marker genes were cloned upstream of the *ef-1α* IG region on the “A” promoter side, as described in Figure 2B. The stable transfection vector also contained the flanking 5′ *ef-1α* ORF and 3′ *ef-1α* ORF sequences (Fig. 2B) for integration into the *ef-1α* locus of the *B. bovis* genome (Fig. 2C). Consequently, the resulting transfection plasmid named *pEf-msa-1-Bm86ep-gfp-bsd* contains the full size ∼1.4 Kb *ef-1α* IG region controlling expression of the *gfp-bsd* gene and the chimeric *msa-1-bm86ep* gene (Fig. 2B).

Plasmid *pEf-msa-1-Bm86ep-gfp-bsd*, and control plasmid *pBluescript* (*pBS*) were separately electroporated into Mo7 *B. bovis* infected RBCs (iRBCs), and then selected with 4 µg/ml blasticidin starting eight hours post-electroporation. After eleven days under selection, no parasites were detected by microscopic analysis in the culture wells containing control *pBS*-and *pEf-msa-1-Bm86ep-gfp-bsd* electroporated parasites, while after fifteen days of selection, the percentage parasitized erythrocytes (PPE) in the *pEf-msa-1-Bm86ep-gfp-bsd* electroporated parasites began to increase (Fig. S1A). In addition, GFP fluorescence was detected in blasticidin resistant parasites transfected with *pEf-msa-1-Bm86ep-gfp-bsd*, whereas neither parasites nor green fluorescence were detectable in cultures selected with blasticidin from parasites transfected with the control plasmid *pBS* (Fig. S1B). The *pEf-msa-1-Bm86ep-gfp-bsd* transfected parasite line emerging from these cultures was designated Tf-Bm86ep-gfp-bsd.

**Figure 3:**
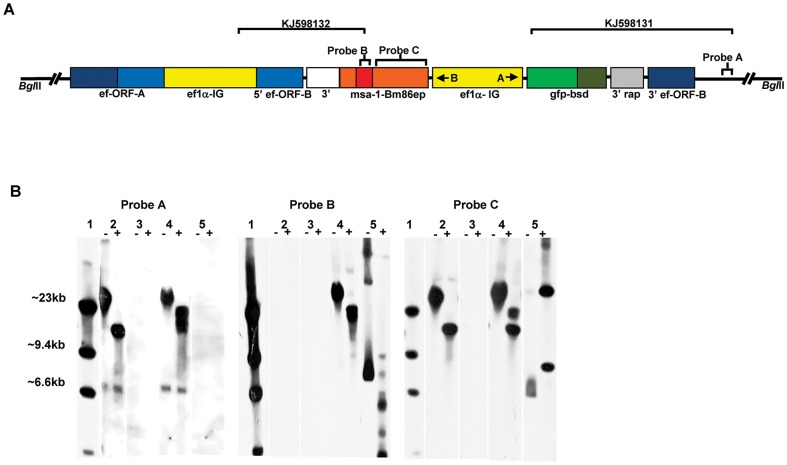
Integration of transfected sequences into the genomic *ef-1α* locus of parasite line Tf-Bm86ep-gfp-bsd. **A**. Schematic of the proposed integration site of *pEf-msa-1-Bm86ep-gfp-bsd* into the *ef-1α* ORF of the *B. bovis ef-1α* locus. Large brackets with GenBank accession numbers KJ598132 and KJ598131 represent the regions amplified and sequenced to demonstrate integration of transfected genes into *B. bovis ef-1α* B ORF locus. Smaller brackets represent the regions of hybridization for southern blot probes A-C. Relative positions of *Bgl*II restriction sites are shown at each end of the schematic. **B**. Shows the results of southern hybridization analysis using dig-labeled probes against *ef-1α* (Probe A), *Bm86ep* (Probe B), and *msa-1* (Probe C) specific dig-labeled specific probes. Each DNA sample was analyzed using undigested (-) or digested (+) *Bgl*II restriction enzyme. Sample order is as follows: 1) dig-labeled DNA ladder, 2) Mo7 wild type parasites, 3) plasmid *pBS*, 4) Tf-Bm86ep-gfp-bsd, 5) *pEf-msa-1-Bm86ep-gfp-bsd*.

To determine if the exogenous genes successfully integrated in the targeted *ef-1α* ORF of the *B. bovis ef-1α* locus in transfected parasites, we performed sequence and Southern blot analysis. Two PCR amplicons were sequenced from Tf-Bm86ep-gfp-bsd spanning the ends of integration on both ends of the transfection plasmid (Fig. 3A) (GenBank accession numbers: KJ598132 and KJ598131). Analysis of both PCR amplicons show both genomic and plasmid DNA sequences organized in a fashion that is fully consistent with integration of the transfected genes in the *ef-1α* B-ORF (Fig. 3A). The Tf-Bm86ep-gfp-bsd parasites were further analyzed by Southern blots using three labeled probes. Total DNA from the transfected parasites along with the plasmid constructs were digested with restriction enzyme *Bgl*II. This enzyme does not cut in the transfection plasmids but does generate a 12,431 bp fragment containing the *ef-1α* in wild type Mo7 *B. bovis* parasites (Fig. 3A and Suarez and McElwain 2009). The blots were then hybridized with *ef-1α Bm86ep*, and *msa-1* specific dig-labeled specific probes (Probes A, B and C, respectively) (Fig. 3A). The results of the Southern blots are shown in Figure 3B. Consistently with insertion of the exogenous genes into the *ef-1α* locus as demonstrated by sequencing of the PCR amplicons, there is an upward shift of the fragment hybridizing with the specific *ef-1α* probe due to the expected increase of the *Bgl*II fragment in the Tf-Bm86ep-gfp-bsd parasite line (Fig. 3B, Probe A, lane 4) when compared to the Mo7 wild type (Fig. 3B, Probe A, lane 2). However, this hybridization band is wide and we cannot rule out the presence of other rearrangements of the *ef-1α* locus in transfected parasites. Neither the *pEf-msa-1-Bm86ep-gfp-bsd* or *pBS* plasmid controls hybridized with the *ef-1α* probe, due to the lack of sequence homology in these plasmids (Fig. 3B, Probe A, lanes 3 and 5). In addition, a probe specific to the *Bm86* epitopes hybridizes to a single DNA fragment in the Tf-msa-1-Bm86ep parasite line that is similar in size to that produced by the *ef-1α* probe (Fig. 3A, Probe B, lane 4), indicating co-localization of the transfected gene and the *ef-1α* ORF. The *pEf-msa-1-Bm86ep-gfp-bsd* also hybridizes with the *Bm86ep* probe (Fig. 3B, Probe B, lane 5), but none of the bands co-localize with Tf-Bm86ep-gfp-bsd, suggesting absence of free transfection plasmid in the DNA extract of the parasite line Tf-Bm86ep-gfp-bsd. Finally, when a probe specific for *msa-1* is used, the Mo7 and Tf-Bm86ep-gfp-bsd cell lines all consistently produce a band of the same size indicative of hybridization with the native version of the *msa-1* gene present in these cells (Fig. 3B, Probe C, lanes 2 and 4). Consistently, hybridization of the *msa-1* probe with the Tf-Bm86ep-gfp-bsd cell line also produces an additional larger band that is also similar in size to the *ef-1α* and *Bm86ep* specific probes (Fig. 3B, Probe C, lane 4), again suggesting co-localization. This upper band does not co-localize with any of the bands in *pEf-msa-1-Bm86ep-gfp-bsd* plasmid construct (Fig. 3B, Probe C, lane 5). Collectively, together with sequence analysis of PCR amplicons, the Southern blot results confirmed stable integration of the transfected genes into the *ef-1α* ORF-B of *B. bovis*, and that free plasmids or episomal DNA are not present. The presence of co-migrating unique bands with probes *ef-1α*, *msa-1*, and *Bm86ep*, is consistent with a single site of integration of the exogenous transfected genes.

**Figure 4:**
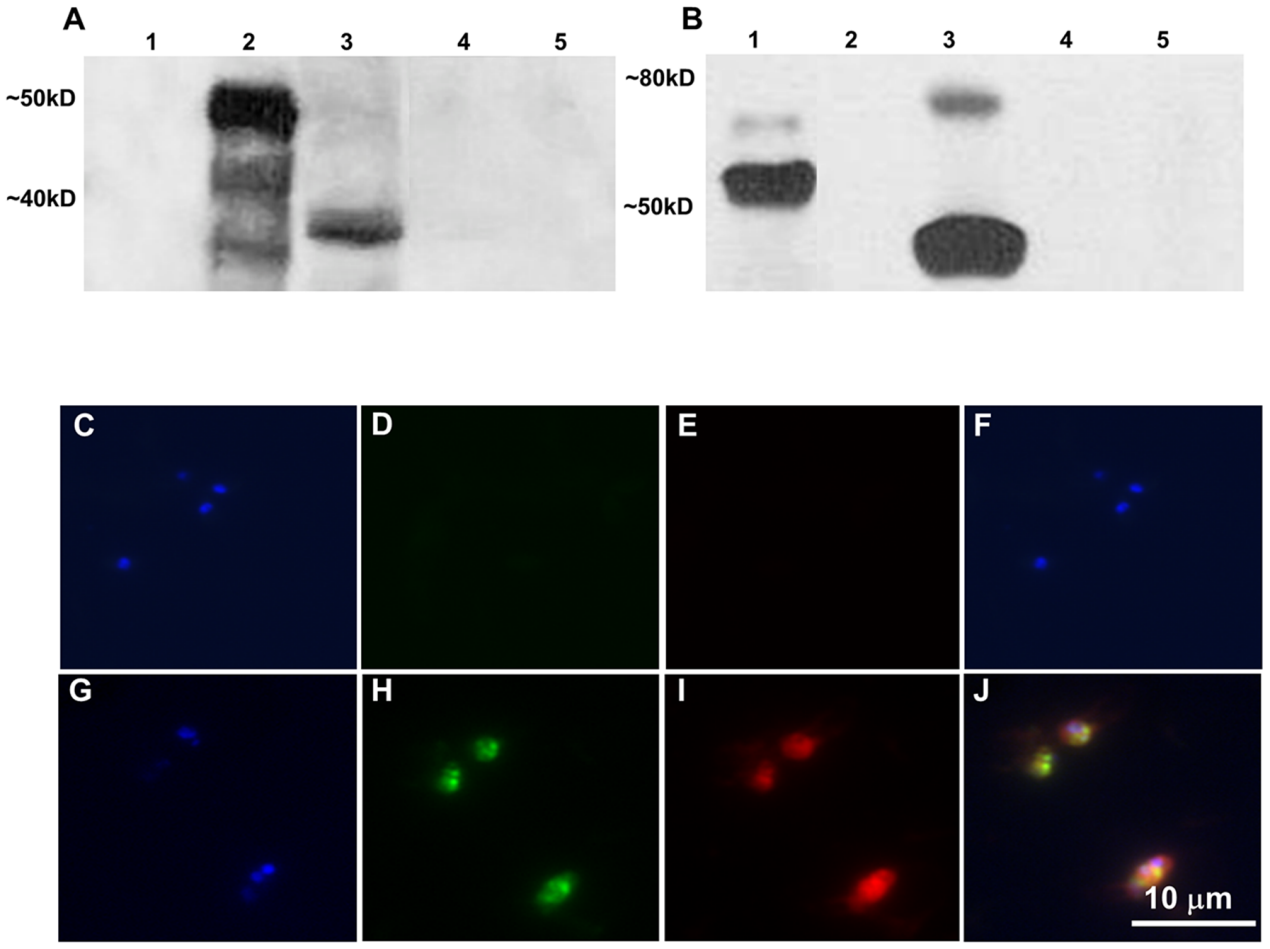
The *gfp-bsd* and *MSA-1-Bm86ep* transgenes are co-expressed in transfected parasites. **A–B** Are the results from immunoblots using GFP (A) and Bm86 (B) specific antibodies. Lanes 1–5 represent recombinant MSA-1-BM86ep, recombinant GFP-BSD, whole cell lysate from the Tf-Bm86ep-gfp-bsd parasite line, whole cell lysate from Mo7 wild type, and non-infected RBC lysate control respectively. Molecular masses are indicated on the left of each panel. **C–J** Show results of fixed cell immunofluorescence using *B. bovis* Mo7 (C–F) and Tf-Bm86ep-gfp-bsd (G–J) infected erythrocytes from cultured cell lines. Parasites were stained with DAPI nucleic acid stain (C–G), GFP antibody labeled with Alexa Flour 488 (D–H), and Bm86ep antibody labeled with Alexa Flour 555 (E–I). Images were visualized using epifluorescence microscopy for each label, and a merged image was created (F–J). A 10 micron size bar is included in the bottom right panel.

To determine whether transfected parasites are able to express the exogenous genes, we used immunoblot and fixed immunofluorescence analysis. Anti-GFP antibodies bound mainly to the predicted 50 kDa recombinant GFP-BSD-thiofusion control, (Fig. 4A, lane 2) and to a 38 kDa protein in Tf-Bm86ep-gfp-bsd, which is the predicted size of the GFP-BSD protein (Fig. 4A, lane 3). However, neither recombinant MSA-1-Bm86ep, Mo7-infected lysate, nor non-infected bovine erythrocyte lysate showed reaction to GFP antibodies as shown in lanes 1, 4, and 5 respectively (Fig. 4A). An approximately 44 kDa protein, consistent with the predicted size of the MSA-1-Bm86ep chimera, was bound by anti-Bm86 antibodies in the Tf-Bm86ep-gfp-bsd parasite line (Fig. 4B, lane 3). The anti-Bm86 antibodies also bound the 56 kDa recombinant MSA-1-Bm86ep positive control (Fig. 4B, lane 1) while none of the recombinant GFP-BSD, Mo7, or uninfected RBC (nRBC) protein lysates showed reactivity with anti-Bm86 as seen in lanes 2, 4, and 5 respectively (Fig. 4B).

Fixed cell immunofluorescence assays were performed using parasites from Mo7 and Tf-Bm86ep-gfp-bsd incubated with anti-GFP tagged with Alexa Fluor 488 and anti–Bm86 tagged with Alexa Fluor 647, and stained with DAPI. The immunofluorescence results are shown in panels C through J in Figure 4. Mo7 DAPI stained cells (Fig. 4C) showed no fluorescent signals from GFP (Fig. 4D) or Bm86ep (Fig. 4E). However, Tf-Bm86ep-gfp-bsd DAPI stained cells (Fig. 4G) showed co-expression of GFP (Fig. 4H) and Bm86ep (Fig. 4I). Merged images of immunolabeled parasites are shown in Figure 4F and J. Taken together, these results demonstrate that the transfected parasites express full size versions of the transfected genes, and co-express both the MSA-1-Bm86ep chimera and GFP-BSD exogenous proteins within single cells of the transfected Tf-Bm86ep-gfp-bsd parasites.

### 2.3 Surface expression of Bm86 epitopes by transfected Tf-Bm86ep-gfp-bsd *B. bovis* merozoites

**Figure 5:**
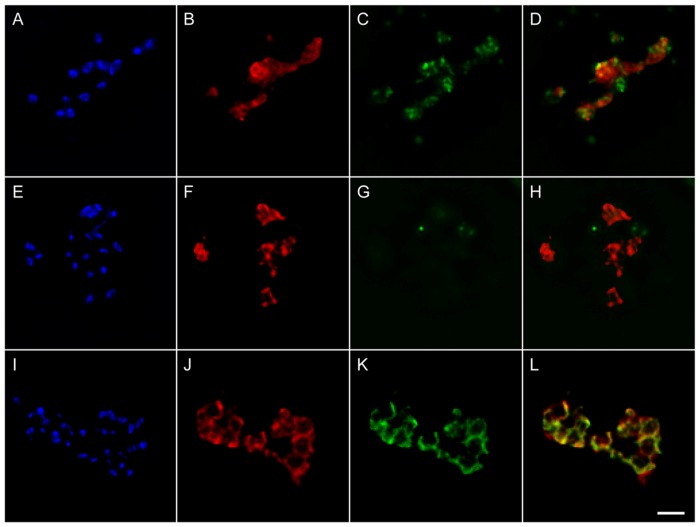
Bm86 epitopes are expressed on the surface of Tf-Bm86ep-gfp-bsd extraerythrocytic merozoites. **A–D** represent permeabilized extraerythrocytic merozoites stained with DAPI (A), incubated with Bm86ep antibody labeled with Alexa Flour 647 (B), and GFP antibody labeled with Alexa Flour 488 (C). A merged image of panels B and C is shown in panel D. **E–H** represents identical staining procedures applied to non-permeabilized cells. **I–L** represent non-permeabilized extraerythrocytic merozoites stained with DAPI (I), and incubated with Bm86ep antibody labeled with Alexa Flour 647 (J), and the MSA-1 mAb Babb35 labeled with Alexa Flour 488 (K). A merged image of panels J and K is shown in panel L. A two micron size bar is included on the bottom right panel.

Surface expression of selected *Bm86* B-cell epitopes in the Tf-Bm86ep-gfp-bsd parasite line was then examined by immunofluorescence using permeabilized or non-permeabilized extraerythrocytic merozoites. Parasites were incubated with anti-Bm86, monoclonal BABB35 (immunoreactive with *B. bovis* MSA-1), and/or anti-GFP primary antibodies followed by incubation with Alexa Fluor labeled secondary antibodies (Fig. 5). Permeabilized (Fig. 5A-D) and non-permeabilized (Fig. 5E-H) transfected parasites were incubated with anti-Bm86 and anti-GFP antibodies and subsequently labeled using Alexa Fluor 647 and Alexa Fluor 488, respectively. Labeled slides were then coverslipped using mounting medium containing DAPI to stain the parasites. In DAPI-stained permeabilized cells, strong anti-Bm86 (Fig. 5B) and anti-GFP (Fig. 5C) immunofluorescence were observed. In contrast, identical incubations in non-permeabilized cells (Fig. 5E–H) resulted in immunolabeling for only anti-Bm86 (Fig. 5F) but not for anti-GFP (Fig. 5G). These data are consistent with the expected intra-cellular expression of GFP, a localization that is not accessible to immunolabeling without first permeabilizing the parasite outer membrane, and the targeted expression of Bm86 epitopes on the surface of transfected parasites, as evidenced by immunolabeling regardless of the integrity of the outer parasite membrane. To further confirm surface expression of the Bm86 epitopes in transfected parasites, we also demonstrated the identical pattern of immunolabeling of MSA-1, a well-known constitutively expressed surface protein of these parasites (Fig. S2). Importantly, mAb BABB35 does not recognize the MSA-1-Bm86ep chimera protein expressed by Tf-Bm86ep-gfp-bsd parasites since the coding region of the MSA-1 epitope recognized by BABB35 was replaced by the Bm86 coding sequence. A final demonstration of co-surface expression of the MSA-1-Bm86 chimera protein with the native expression of MSA-1, simultaneous incubations were performed using non-permeabilized cells with both anti-Bm86 (Fig. 5J) and BABB35 (Fig. 5K) antibodies showing similar surface localization of both proteins (Fig. 5L). Spatial co-occurrence was objectively measured for the entire de-convolved image stacks of Figure 5J and 5K, and revealed that ∼82% of BM86ep co-occurred in the compartment containing MSA-1 and ∼91% of MSA-1 co-occurred in the compartment containing Bm86ep. Visual inspection of the resulting threshold image stacks confirmed spatial co-occurrence of MSA-1 and Bm86ep along the entire surface of free merozoites (Fig. S3). Collectively, the results demonstrate that the Bm86 epitopes of the MSA-1-Bm86ep chimeric protein are exposed on the surface of transfected Tf-Bm86ep-gfp-bsd merozoites in a fashion that is similar to the surface exposed MSA-1.

## Discussion

A goal is to use *B. bovis* transfected parasites as platforms for the delivery of protective exogenous antigens. Ideally, such transfected parasites should be able to express a selectable marker and the antigen of interest independently. In addition, the antigen of interest should be expressed in a configuration that favors proper presentation to the immune system of the host. Limitations of previously developed transfection constructs include the expression of only a single transgene of uncertain cellular localization [8].

Inclusion of an additional expression site requires the addition of an extra independent promoter in the transfection construct. Promoter activities of the *ef-1α* IG region in *B. bovis* has been previously tested in transiently transfected *B. bovis* using two IG-derived fragments, “IG-A” and “IG-B”, controlling expression of a luciferase reporter gene. High levels of expression of luciferase were shown to be promoted by both fragments, which supported the presence of two independent promoters in the IG-region between the two *ef-1α* genes [9]. These preliminary data provided strong rationale for the use of the complete 1.4 Kb *ef-1α* IG region as the source of a bi-directional promoter, but bi-directional promoter activity of the full 1.4 Kb *ef-1α* IG region was so far not conclusively demonstrated. Use of the full size 1.4 Kb *ef-1α* IG region could in theory favor biased promoter activity, or the activity of each promoter could be diminished due to competition for RNA polymerases and factors required for transcription. Thus to address these issues, we first studied whether the IG region of the *ef-1α* locus contains a bidirectional promoter by testing expression of the luciferase gene in a transient transfection system. The results comparing expression of a single luciferase gene cloned at either end of the IG region are consistent with bidirectional promoter activity using the full IG region. This was further confirmed by the significant increase in luciferase activity when two functional luciferase genes were cloned in the correct orientation into both expression sites in the complete IG region. This effect was not caused by increased number of transfected parasites nor by the number of gene copies of luciferase per transfected cell. Thus, the increased luciferase activity suggests simultaneous bidirectional expression of the two genes. Taken together, any competition for transcription factors does not appear to compromise expression on either side of the promoter to a significant extent. Importantly, the transient transfection results using the full size *ef-1α* IG region allowed development of a simple head to head tandem arrangement transfection vector that includes both a selectable marker gene in one of the expression sites and another gene of interest in the other.

Targeting surface expression of known B-cell epitopes may optimize presentation to the immune system of the host for the induction of antibody responses. To attempt surface expression of exogenous genes in transfected parasites, sequences from the surface expressed *B. bovis* antigen MSA-1 were combined in a chimeric gene with selected sequences derived from Bm86 which have been previously shown to encode protective B-cell epitopes [13], [14]. The selection of MSA-1 as the frame of the chimeric gene was based on previous studies which characterized it as a strongly immunodominant antigen containing a leader sequence, body, hypervariable region (HVR), and glycosylphosphatidylinositol (GPI) anchor [20], [21], [24], [25]. Furthermore, the neutralization sensitive, surface reactive monoclonal antibody BABB35 binds a B-cell epitope present in the HVR of MSA-1, indicating that the HVR of MSA-1 is surface exposed and probably required for erythrocyte invasion [20], [21], [22], [24], [25]. Our experimental design used the immunodominant MSA-1 as a scaffold in a chimeric construct where the most highly antigenic and surface exposed region of this molecule is replaced by sequences encoding previously defined protective Bm86 derived B-cell epitopes. The expectation was that this strategy would result in surface expression of Bm86 epitopes. This MSA-1-Bm86ep chimera was developed as a model to test this concept by incorporating it into a transfection plasmid containing the bidirectional *ef-1α* promoter for expression in *B. bovis* parasites.

Immunoblots and immunofluorescence analysis demonstrated co-expression of both transfected exogenous proteins by single cells, consistent with the proposed bidirectional promoter activity by the full size *ef-1α* IG region. Furthermore, the pattern of fluorescence on permeabilized and non-permeabilized extracellular merozoites demonstrates intracellular expression of the GFP-BSD protein and surface exposure of the Bm86 epitopes in transfected *B. bovis* parasites. Significantly, the majority of fluorescent patterns co-occur when Bm86ep and MSA-1 antibodies, which should recognize only wild type MSA-1, are used together, further demonstrating surface localization of these epitopes of non-permeabilized merozoites.

In summary, a novel transfection system using a bidirectional promoter that increases the capacity for expression of exogenous genes and allows surface expression of selected epitopes was developed and tested. The data demonstrate the feasibility for stable integration and expression of large transgenes in the *ef-1α* locus of *B. bovis*, and show that replacing the sequences encoding the MSA-1 HVR with exogenous sequences in the full *msa-1* gene results in exposure of foreign epitopes in the surface of transfected parasites.

## Materials and Methods

### 4.1. Parasites

The Mo7 biological clone of *B. bovis* was derived by limiting dilution of the Mexico strain as described [20], [26] and was maintained as a cryopreserved stabilate in liquid nitrogen [27]. Parasites were grown in long term microaerophilous stationary-phase culture by previously described techniques [28].

### 4.2 Analysis of bidirectional ef-1α IG promoter activity in transiently transfected *B. bovis*

To analyze whether the full length 1.4 Kb *ef-1α* IG region is able to promote co-expression of two distinct genes, three plasmids containing the *ef-1α* IG and luciferase (*luc*) genes were designed. To produce these plasmids, a 1.4 Kb DNA fragment identical to the native *ef-1α* IG was designed and synthesized (GeneScript). The resulting synthetic DNA fragment was then cloned in both possible orientations into a *Hind*III and alkaline phosphatase treated plasmid *p40-15-luc* which contains the luciferase gene for reporter capability in the *EcoR*I site, and the 3′ *rap-1* gene to stop transcription in the *Pst*I site as previously described [9]. Then a *luc-3′rap-1* fragment was generated using *Kpn*I*-luc-F* (5′-atccgcggtaccatggaagacgccaaaaacataaag-3′) and *Kpn*I*-trancon-R* (5′-ggtacctcctttagtgaggttcacg-3′) primers and cloned in both direction into the *Kpn*I site of the two previously produced plasmids. Collectively this resulted in three plasmids containing the synthetically produced *ef-1α* IG with 1) functional luciferace genes positioned on either side of the promoter named *pEfIgA&Bluc*, 2) one functional luciferace gene positioned upstream and a non-functional luciferace gene downstream of the promoter named *pEfIgBluc*, and 3) one functional luciferace gene positioned downstream and a non-functional luciferace gene upstream of the promoter named *pEfIgAluc*. These plasmid constructs, along with a plasmid containing *luc* without a promoter, were then electroporated in triplicate into cultured Mo7 *B. bovis* infected erythrocytes as previously described [9]. Parasitemia was monitored by light microscopy from each transfected parasite culture at four and twenty-four hours. Luciferase activity was detected with a Turner Designs TD-20/20 Tube Luminometer for 10 second integrations twenty-four hours post-transfection using the transfected *B. bovis* lysate as previously described [9]. The data was statistically analyzed using a two-sample t-test for differences among the treatment groups. Using the same material from the luciferase assay, a quantitative real-time PCR (qPCR) was standardized to assess the copy numbers of luciferase gene in the transfected *B. bovis* parasites. For the luciferase qPCR, the following primers were designed to amplify a 152 bp fragment: 5′ ggttttggaatgtttact 3′ and 5′ gcgaagaaggagaatag 3′. The qPCR were performed in a CFX96™ Real-Time PCR Detection System using the SsoFast™ EvaGreen® Supermix (Bio-Rad). The cycling conditions consisted of an enzyme activation step of 95°C for 30 seconds followed by 40 cycles of 95°C denaturation for 5 seconds and annealing/extension of 60°C for 5 seconds. Reactions were performed in duplicate in 20 µl using 400 nM of each primer and 2 µl of a 1/20 dilution of DNA samples. The CFX Manager™ Software (Bio-Rad) was used to analyze the qPCR data. Copy numbers of luciferase gene was calculated based on a standard curve as previously described [29]. For the luciferase qPCR, an efficiency of amplification of 100.6% (R^2^ = 0.994 and slop = −3.307) was obtained. Moreover, specificity and analytical sensitivity was assessed by melt curve analyses and standard curve, respectively. Additionally, the copy numbers of *B. bovis msa-1* gene in transfected parasites was also investigated as previously described [29]. Considering that the *B. bovis msa-1* is a single copy gene and each transfection plasmid contains two luciferase genes, total copy numbers of luciferase per transfected parasites was calculated and averaged among the transfection replicates to assess efficacy between the three transfection plasmids. Statistical significance was analyzed using a two-sample t-test for differences among the treatment groups.

### 4.3 Generation of msa-1-bm86ep chimera and plasmid constructs for stable transfection of *B. bovis* infected erythrocytes

To develop the *msa-1-Bm86ep* chimera, gene sequences were designed and synthesized (GeneScript). First, sequences from four specific regions within *Bm86*, base pairs (bp) 60–171, 393–432, 1191–1254 and 1546–1684, which have been previously described as B-cell epitopes [13], [14], were aligned linearly resulting in a gene called *bm86ep*. Next, the chimeric gene *msa-1-bm86ep* was designed by placing the sequence from bp 1-600 of *msa-1* immediately upstream of *bm86ep* sequences, and the sequence from bp 900–990 of *msa-1* immediately downstream of the *bm86ep* sequences. The sequence of the chimeric gene was submitted to GeneBank (KJ598130). The region encompassing bp 601 to bp 900 of the *msa-1* gene encodes for the hypervariable region of MSA-1, including the region recognized by mAb BABB35, thus the chimeric MSA-1Bm86ep protein does not bind mAb BABB-35 (data not shown). *Sac*II restriction sites were added to the ends the chimeric sequence and the gene was synthesized into *EcoR*I restriction sites of the *pUC57* plasmid vector (GeneScript). Synthetic *msa-1-bm86ep* was then ligated into the *Sac*II site of the elongation factor transfection vector *pEf-Sac*II*-gfp-bsd*, which was constructed as follows: *gfp-bsd* was amplified from *pTracer* (Invitrogen) using Tracer-*EcoR*V*-gfp-F* (5′-cgtcgtgatatcatggcctccaaaggagaac-3′) and *EcoR*V*-bsd-R* (5′-taatgtgatatcgccctcccacacataaccagag-3′) primers, and was ligated into *pBlueScript* treated with *EcoR*V and alkaline phosphatase, resulting in *p-gfp-bsd*. The 5′ *ef-1α* ORF was then amplified from *B. bovis* gDNA using *Sac*I*-Ef-orf-F1* (5′-ctgacggagctcatgccgaagactcac-3′) and *Sac*I*-Ef-orf-R1* (5′-cagctggagctcatctgatcaagggcctcgacc-3′) primers and ligated into the *Sac*I and alkaline phosphatase treated *p-gfp-bsd*, resulting in *p-5′eforf-gfp-bsd*. Next, the 3′ *ef-1α* ORF and a 3′ *rap-1* region were amplified from *p-2-1-130* [8] using *Apa*I*-rap-int-F* (5′-gcatgcagggccccaatttgcgcagatgaagaat-3′) and *Apa*I*-Ef-orf-R2* (5′-gcactagggccctcttagcagccttttgggcagac-3′) primers and ligated into an *Apa*I and alkaline phosphatase treated *p-5′eforf-gfp-bsd*, resulting in *p-5′eforf-gfp-bsd-3′eforf*. The 1.4 Kb *ef-1α* IG synthetic gene representing the full *B. bovis ef-1α* intergenic region [9] was ligated into a *Sma*I and alkaline phosphatase treated *p-5′eforf-gfp-bsd-3′eforf*, resulting in *pEf-Sac*II*-gfp-bsd*. The final transfection vector was named *pEf-msa-1-Bm86ep-gfp-bsd*. Twenty micrograms of this plasmid, along with *pBlueScript* control, were separately diluted into 25 µl Cytomix and electroporated with 75 µl of approximately 20% *B. bovis* infected red blood cells (RBCs) as previously described [8] creating the transfected Tf-Bm86ep-gfp-bsd and Tf-pBS *B. bovis* cell lines. Six hours after transfection the cell lines were selected with 4 µg/ml blasticidin, and the growth rates were recorded daily using blood smears and light microscopy.

### 4.4 Genetic analysis

Parasite gDNA was isolated from Tf-Bm86ep-gfp-bsd and Mo7 wild-type cell lines for sequencing of PCR integration amplicons and Southern hybridization analyses. PCR amplification was conducted using primer sets *EcoRV-gfp-F* with *UPS-Ef-probe-R* (5′-cacgcgcaatatcacagttccatc-3′) and *Bm86ep-F* (5′-gggaacgagttctgtcgcaacgc-3′) with Ef-pr-F8 (5′-gtctttataacttaataaagtaattcc-3′). The resulting PCR products were then confirmed by gel electrophoresis, cloned into pCR 2.1 Topo Vector (Invitrogen), sequenced in full using standard techniques, and submitted to GeneBank (Accession numbers: KJ598132 and KJ598131). For Southern hybridization analysis, total DNA from *B. bovis* merozoites was digested with *Bgl*II, electrophoresed, transferred to ZetaProbe nylon membranes, and hybridized as previously described [8]. Digoxigenin-labeled probes representing the complete *bm86ep* synthetic gene, 300 bp regions upstream of the *ef-1α* locus, and complete *msa-1* were prepared by PCR amplification using a PCR Dig-Probe Synthesis kit as recommended by the manufacturer (Boehringer–Roche). The *bm86ep* probe was prepared by PCR with primers *Bm86ep-F* (5′-gggaacgagttctgtcgcaacgc-3′) and *Bm86ep-R* (5′-cgatatgatatcaggacacttgcattctgc-3′). The *ef-1α* probe was prepared by amplification of *B. bovis* gDNA with primers *UPS-Ef-probe-F* (5-acagaataaatatgtttaaaac-3′) and *UPS-Ef-probe-R*. The *msa-1* probe was prepared by amplification of Mo7 genomic DNA with primers *msa1-Topo-F* (5′-atggctacgtttgctcttttcatttc-3′) and *msa1-Topo-R* (5′-aaatgcagagagaacgaagtagc-3′).

### 4.5 Immunoblot analysis

To confirm the expression of correctly sized GFP-BSD and MSA-1-Bm86ep fusion proteins, merozoites of Tf-Bm86ep-gfp-bsd cell line were subjected to SDS-PAGE and analyzed by western blot. The samples used were derived from Tf-Bm86ep-gfp-bsd and Mo7 wild-type whole culture lysates and recombinant Thio-GFP-BSD and Thio-MSA-1-Bm86ep fusion proteins and transferred in triplicate to nitrocellulose membranes. The first membrane was probed with a 1∶500 dilution of polyclonal anti-Bm86ep primary antibodies and a 1∶5,000 dilution of goat anti-rabbit-immunoglobulin peroxidase conjugate (Life Biosciences) of secondary antibodies. The second membrane was probed with a 1∶1000 dilution of polyclonal anti-GFP primary antibodies and a 1∶5,000 dilution of goat anti-rabbit-immunoglobulin peroxidase conjugate (Life Biosciences) secondary antibodies. The third membrane was probed with a 2 µg/ml dilution of monoclonal anti-MSA-1 monoclonal primary antibodies (BABB35) (20) and a 1∶5,000 dilution of goat anti-mouse-immunoglobulin peroxidase conjugate (Life Biosciences) secondary antibodies.

### 4.6 Fixed cell immunofluorescence assay

The GFP fluorescence of the transfected parasite lines was followed using live cells from the blasticidin selected cultures. Once GFP fluorescence was detected in the Tf-Bm86ep-gfp-bsd cell line, a fixed cell immunofluorescence assay was used to detect simultaneous expression of GFP and Bm86ep. Infected RBCs from Tf-Bm86ep-gfp-bsd, and Tf-pBS were washed once and diluted 1∶10 in 3% bovine serum albumin (BSA)-phosphate buffered saline (PBS). These cells were used to make blood smears which were fixed with 2% paraformaldehyde for 5 minutes and then permeablized with Triton X-100 0.1% for 10 minutes. Next, the slides were incubated with 10% BSA-PBS blocking solution for 10 min and incubated with a 1∶500 dilution of anti-GFP monoclonal antibody (Invitrogen), and a 1∶500 dilution of anti-Bm86ep antibodies for 1 hour. Identically produced negative controls were performed using pre-immune rabbit serum instead of the primary antibodies. All samples were washed twice in PBS and incubated with goat-anti-mouse Alexa Fluor 488 and goat-anti-rabbit Alexa Fluor 555 as the secondary antibodies (Invitrogen). Finally, samples were washed twice in PBS, and one drop of ProLong Gold antifade reagent with DAPI (Invitrogen) was added to each sample. These samples were visualized with epifluorescence microscopy to produce a merged image.

### 4.7 Immunofluorescence of extraerythrocytic merozoites

Merozoites were isolated from Tf-Bm86ep-gfp-bsd and Mo7 cell lines by centrifugation of the supernatant two times at 400 RCF to remove the RBC with a final centrifugation at 2,000 RCF to pellet the merozoites. These parasites were washed in 3% bovine serum albumin (BSA) PBS. A portion on the cells were then incubated with 1∶500 10% BSA dilutions with a combination of either 1) anti-Bm86ep and anti-MSA-1 (BABB35), 2) anti-Bm86ep and anti-GFP, or 3) anti-MSA-1 primary antibodies for one hour. The cells were then washed in the PBS two times with a 400 RCF centrifugation and incubated with 1∶1000 10% BSA dilutions of either 1) goat-anti-rabbit Alexa Fluor 647 and goat-anti-mouse Alexa Fluor 488, 2) goat-anti-rabbit Alexa Fluor 647 and goat-anti-mouse Alexa Fluor 488, or 3) goat-anti-mouse Alexa Fluor 647 and anti-GFP conjugated with Alexa Fluor 488 secondary antibodies for 30 minutes. The cells were again washed two times, dried to a slide and mounted with Prolong Gold anti-fade with DAPI. A second set of slides, to control for cell permeablization, were made by first fixing cells on to a slide with 2% paraformaldehyde for ten minutes, and then by incubating with Triton X-100 0.1% in order to permeabilize. These cells were then incubated with either 1) anti-Bm86ep and anti-GFP, or 2) anti-MSA-1 primary antibodies for one hour, washed two times in PBS, and then incubated with 1) goat-anti-rabbit Alexa Fluor 647 and goat-anti-mouse Alexa Fluor 488, or 2) goat-anti-mouse Alexa Fluor 647 and anti-GFP conjugated with Alexa Fluor 488 for one hour. All samples were then independently visualized by epifluorescence microscopy and image processed as follows. Slides were viewed and digitally photographed using an Axio Imager.M1 microscope (Carl Zeiss) equipped with an X-Cite 120 Fl illuminating system (EXFO Photonic Solutions). Digital images were captured using an AxioCam MRm digital camera connected to a desktop computer running AxioVision (version 4.8.1.0). Image stacks were obtained using optimal z-axis spacing [250 nm z-step, Plan-Apochromat 63x/1.4 oil M27 objective (Carl Zeiss Imaging, Inc.)]. Z-stack image files were imported for processing into the ImageJ-based open source processing package Fiji (version 1.48b; http://pacific.mpi-cbg.de/) [30]. Each fluorescence channel was deblurred using the “Parallel Iterative Deconvolution 3D” plug-in (version 1.11; 250 iterations, MRNSD method) and appropriate theoretical point spread functions generated using the “Diffraction PSF 3D” plug-in (version 2). Each figure panel presents pseudo colored fluorescent channels from a single mid-stack slice from representative images. Colocalization analysis was conducted using the “Coloc 2” plugin, and using the deconvolved image stacks of Figure 5, I-K. Since the image stacks include many pixels outside of the centered merozoite cluster (i.e., outside regions of biologic interest), and since inclusion of numerous low-intensity pixels outside regions of biologic interest can negatively impact objective threshold determinations, a masking stack was created to limit subsequent analysis to fluorescent pixels contributed from either channel [31]. This was accomplished by merging histogram-normalized stacks (saturated pixels = 0.1%) from which background had been subtracted (rolling ball radius = 16 pixels), minimum pixel intensity set to 0, and converted to 8-bit. A Gaussian blur (sigma = 2 pixels) was applied to the stack before thresholding (Huang method using stack histogram).The resulting mask stack and the histogram-normalized image stacks (channel 1 = Alexa647; channel 2 = Alexa488) were used as input to the “Coloc 2” plugin. Since we have no reason to suspect that the cell-to-cell expression and surface localization and distribution of these two proteins are stoichiometric, such as might occur with interacting proteins, we chose to evaluate Manders' Colocalization Coefficients (MCCs) by the algorithm of Costes *et al*. (2004) [32]. The resulting MCCs calculated for fluorescence intensities greater than threshold (tM1 and tM2) objectively inform about the fraction of one fluorophore in the compartment containing the other.

## Supporting Information

Figure S1 **Selection of the transfected** ***B. bovis*** **cell line after electroporation of** ***B. bovis*** **merozoites with plasmid** ***pBm86ep-gfp-bsd*** **.** **A**. Growth curve showing selection with blasticidin (4 µg/ml) added 6 hours after electroporation of parasite lines Tf-Bm86ep-gfp-bsd (red) and Tf-pBS transfected control parasites (blue). Days post transfection is indicated along the x axis and percentage parasitized erythrocytes (PPE) along the y axis. **B**. Detection of GFP protein by live cell epifluorescence microscopy. Blasticidin selected parasites of the Tf-Bm86ep-gfp-bsd and control Tf-pBS parasites are shown. A five micron size bar is included in the right panel.
(TIF)

Figure S2 **MSA-1 epitopes are expressed on the surface of Tf-Bm86ep-gfp-bsd extraerythrocytic merozoites.** **A–D** represent permeabilized extraerythrocytic merozoites stained with DAPI (A), and incubated with MSA-1 monoclonal antibody Babb35 labeled with Alexa Flour 647 (B), and GFP antibody labeled with Alexa Flour 488 (C). A merged image of panels B and C is shown in panel D. **E–H** represents identical staining procedures as above but applied to non-permeabilized cells. A two micron size bar is included on the bottom right panel.
(TIF)

Figure S3 **Z-stack montage demonstrating co-occurrence of BM86 and MSA-1 on the surface of cell-free merozoites.** Objective analysis was conducted on the de-convolved image stacks of a cluster of cell-free merozoites fluorescently labeled using antibodies to BM86 and MSA-1 in the absence of permeabilization, a condition that labels only surface epitopes of intact organisms (slice 18 of these stacks are shown in Figure 5I–L). The algorithm of Costes *et al*. (2004) was used to determine Manders' Colocalization Coefficients for threshold fluorescences as determined by the algorithm. In this montage of the merged threshold image stacks, spatially independent BM86 fluorescence is colored red, spatially independent MSA-1 is colored green, and spatial co-occurrence is the color yellow (the sum of red and green). Easily appreciated in this montage scanning through each level (250 nm z-steps) of this cluster of cell-free merozoites, the great majority of each labeled protein co-occurred in the merozoite surface compartment containing the other protein. Thus ∼82% of BM86 co-occurred in the compartment containing MSA-1 and ∼91% of MSA-1 co-occurred in the compartment containing BM86. A two micron size bar is included on the bottom right panel.
(TIF)
