# Supplementary figures and images for: Targeted Surface Expression of an Exogenous Antigen in Stably Transfected Babesia bovis
Source: PLoS One. 2014 May 19;9(5):e97890. doi: 10.1371/journal.pone.0097890 (PMC4026526; doi:10.1371/journal.pone.0097890)

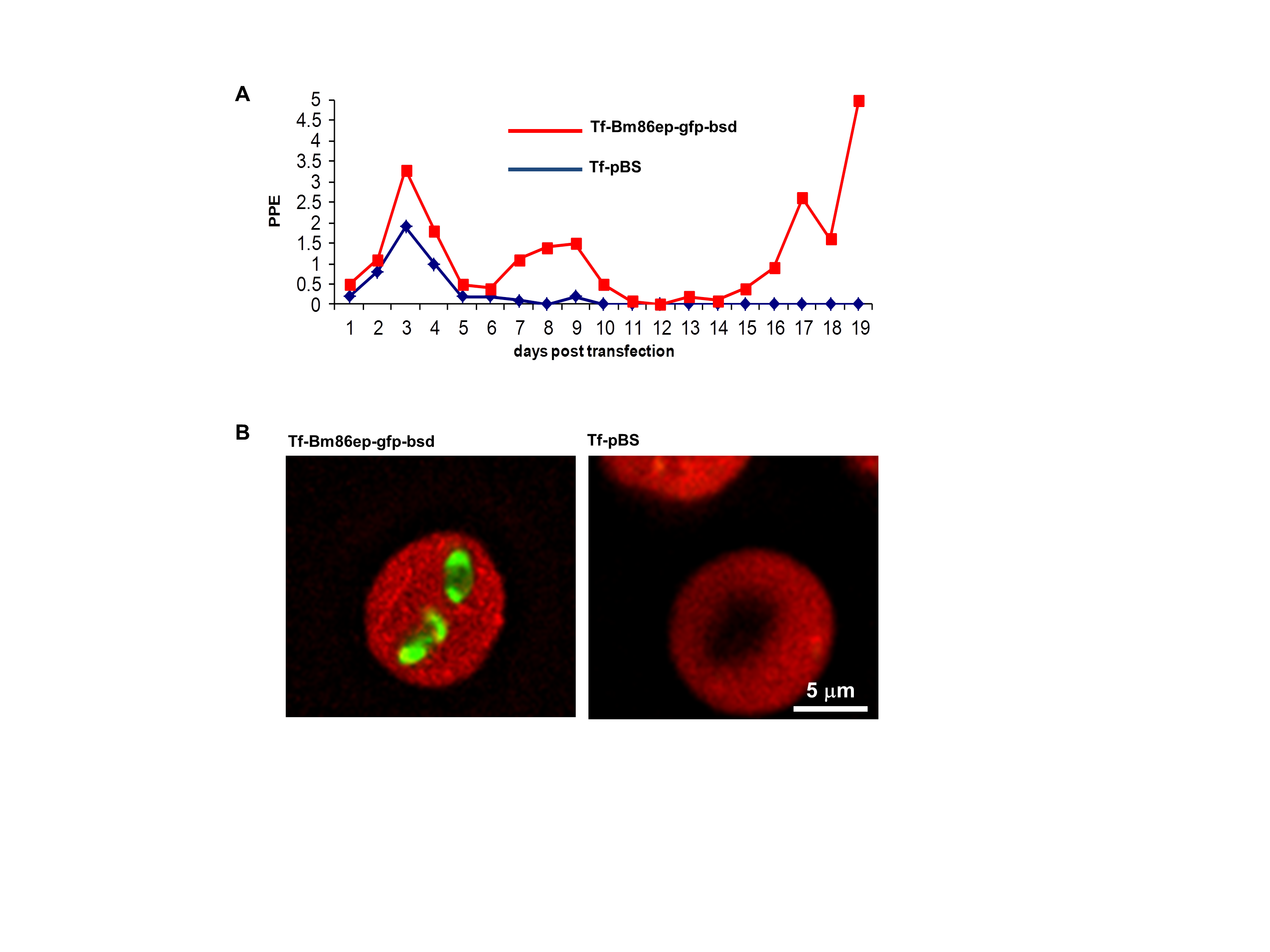

Supplement: Figure S1 — Selection of the transfected B. bovis cell line after electroporation of B. bovis merozoites with plasmid pBm86ep-gfp-bsd . A. Growth curve showing selection with blasticidin (4 µg/ml) added 6 hours after electroporation of parasite lines Tf-Bm86ep-gfp-bsd (red) and Tf-pBS transfected control parasites (blue). Days post transfection is indicated along the x axis and percentage parasitized erythrocytes (PPE) along the y axis. B. Detection of GFP protein by live cell epifluorescence microscopy. Blasticidin selected parasites of the Tf-Bm86ep-gfp-bsd and control Tf-pBS parasites are shown. A five micron size bar is included in the right panel. (TIF) [file pone.0097890.s001.tif]

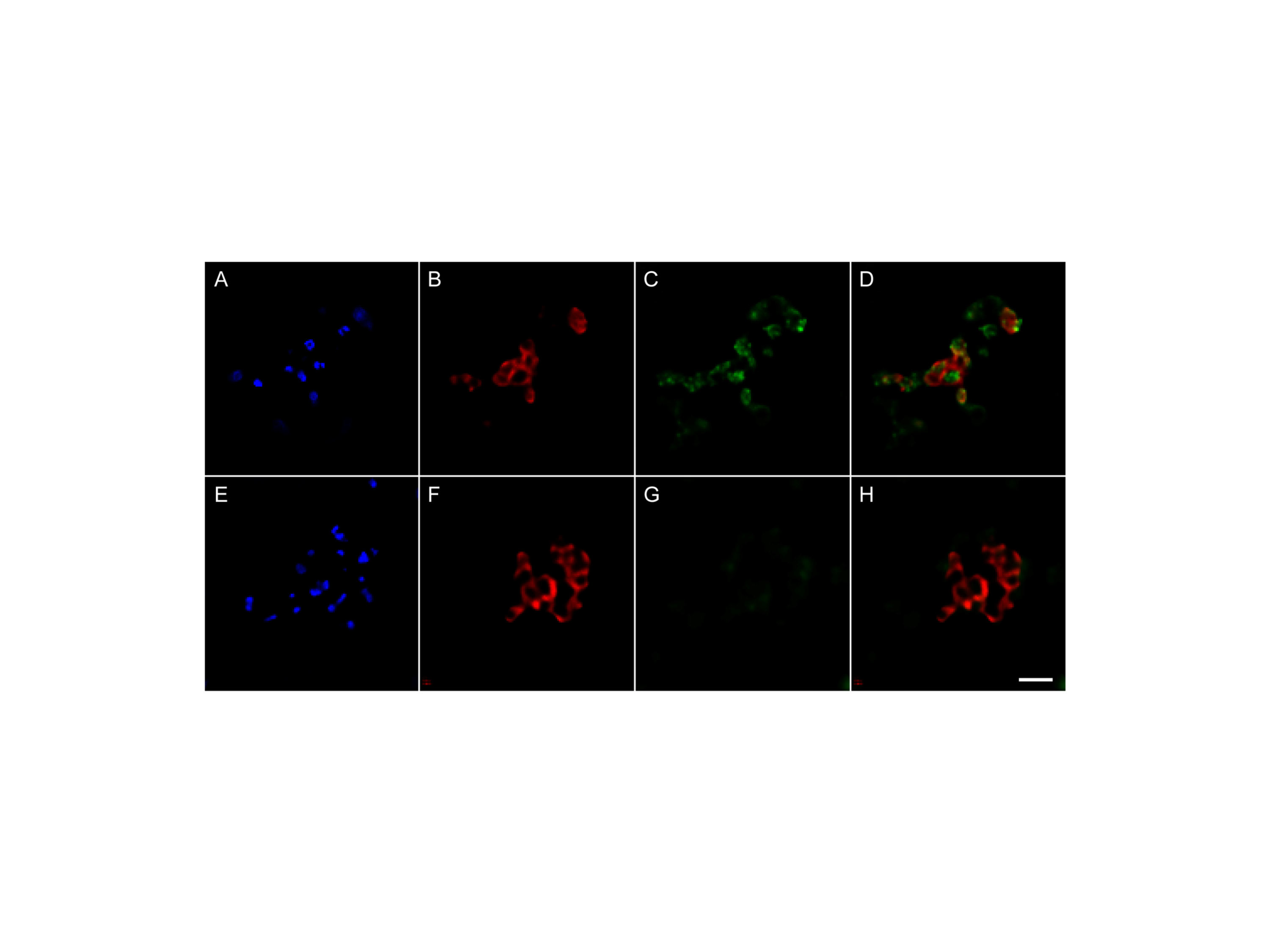

Supplement: Figure S2 — MSA-1 epitopes are expressed on the surface of Tf-Bm86ep-gfp-bsd extraerythrocytic merozoites. A–D represent permeabilized extraerythrocytic merozoites stained with DAPI (A), and incubated with MSA-1 monoclonal antibody Babb35 labeled with Alexa Flour 647 (B), and GFP antibody labeled with Alexa Flour 488 (C). A merged image of panels B and C is shown in panel D. E–H represents identical staining procedures as above but applied to non-permeabilized cells. A two micron size bar is included on the bottom right panel. (TIF) [file pone.0097890.s002.tif]

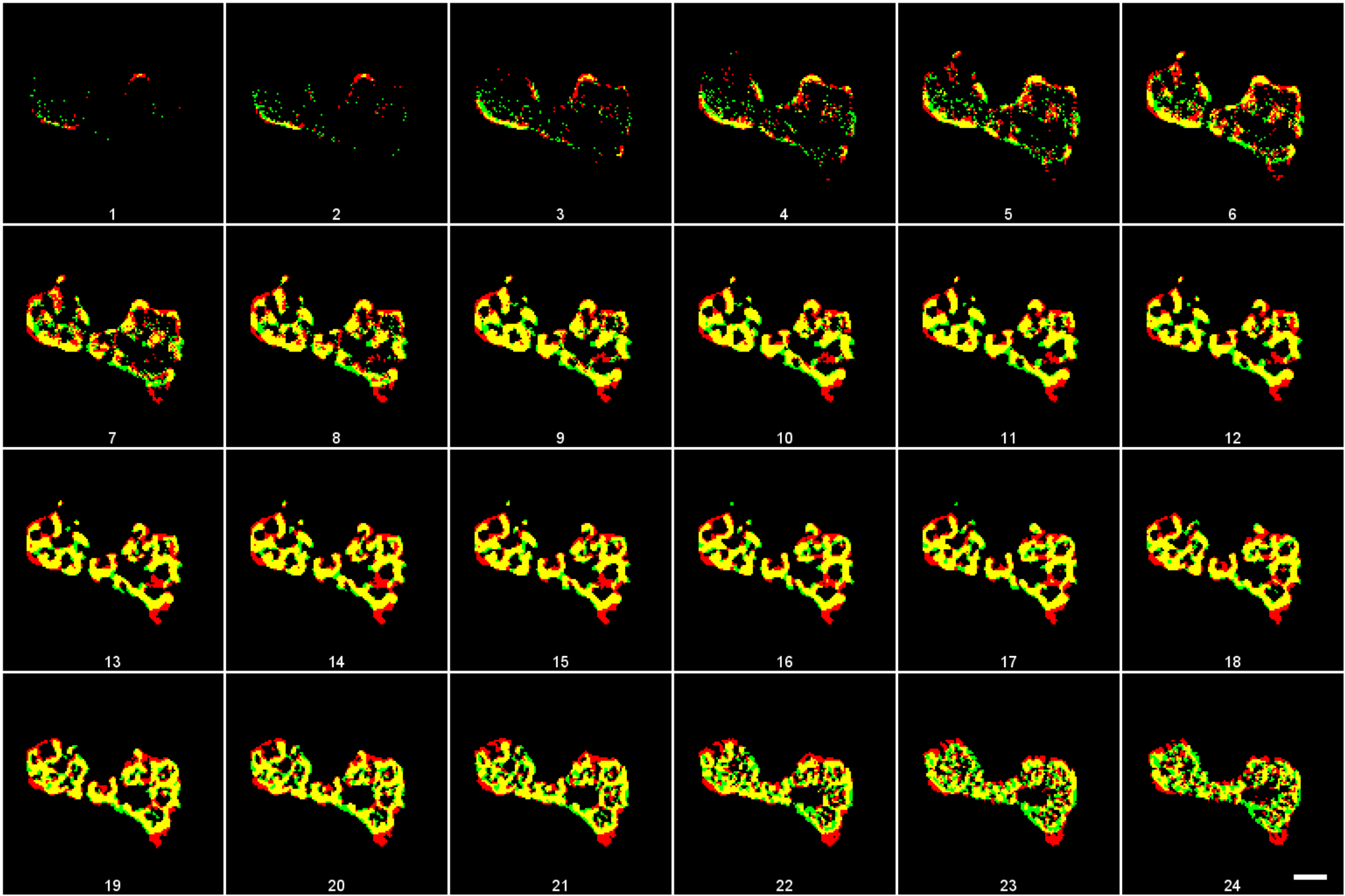

Supplement: Figure S3 — Z-stack montage demonstrating co-occurrence of BM86 and MSA-1 on the surface of cell-free merozoites. Objective analysis was conducted on the de-convolved image stacks of a cluster of cell-free merozoites fluorescently labeled using antibodies to BM86 and MSA-1 in the absence of permeabilization, a condition that labels only surface epitopes of intact organisms (slice 18 of these stacks are shown in Figure 5I–L). The algorithm of Costes et al. (2004) was used to determine Manders' Colocalization Coefficients for threshold fluorescences as determined by the algorithm. In this montage of the merged threshold image stacks, spatially independent BM86 fluorescence is colored red, spatially independent MSA-1 is colored green, and spatial co-occurrence is the color yellow (the sum of red and green). Easily appreciated in this montage scanning through each level (250 nm z-steps) of this cluster of cell-free merozoites, the great majority of each labeled protein co-occurred in the merozoite surface compartment containing the other protein. Thus ∼82% of BM86 co-occurred in the compartment containing MSA-1 and ∼91% of MSA-1 co-occurred in the compartment containing BM86. A two micron size bar is included on the bottom right panel. (TIF) [file pone.0097890.s003.tif]
